# Low-cost informational intervention reduced drinking water arsenic exposure in Bangladesh

**DOI:** 10.1093/pnasnexus/pgac284

**Published:** 2023-03-27

**Authors:** Raghav R Reddy, Grace A van Velden, Md Joynul Abedin, Md Rezaul Karim, Kim F Hayes, Arun Agrawal, Lutgarde Raskin

**Affiliations:** Department of Civil and Environmental Engineering, University of Michigan, Ann Arbor, MI 48109,US; Department of Civil and Environmental Engineering, University of Michigan, Ann Arbor, MI 48109,US; School of Public Health, University of Michigan, Ann Arbor, MI 48109,US; Asia Arsenic Network, 7400 Jessore, Bangladesh; Asia Arsenic Network, 7400 Jessore, Bangladesh; Department of Civil and Environmental Engineering, University of Michigan, Ann Arbor, MI 48109,US; School of Environment and Sustainability, University of Michigan, Ann Arbor, MI 48109,US; Department of Civil and Environmental Engineering, University of Michigan, Ann Arbor, MI 48109,US

## Abstract

Thirty million Bangladeshis continue to drink water with unacceptable levels of arsenic (>10 μg/L), resulting in a large public health burden. The vast majority of the Bangladeshi population relies on private wells, and less than 12% use piped water, increasing the complexity of mitigation efforts. While mass testing and informational campaigns were successful in the early 2,000 s, they have received little attention in recent years, even though the number of wells in the country has likely more than doubled. We investigated the effect of a low-cost (<USD 10/household) informational intervention on reducing arsenic exposure through a randomized control trial design. The sample size was ∼10% of the study area households, and the intervention provided exposure awareness material, the arsenic concentration of the household's drinking water, and information about alternate water sources nearby with improved water quality. The informational intervention lowered household arsenic exposure (*P* = 0.0002), with an average reduction in arsenic levels of ∼60%. Approximately one third of the study households requested to test an additional water source at no cost. Providing the intervention a second time led to more households changing their water source but did not further reduce exposure (*P* = 0.39). Our study establishes a causal relationship between the informational intervention and the observed reduction in household arsenic exposure. Our findings demonstrate that water testing and recommendations for accessing improved water provide an immediate, effective, and inexpensive means of reducing the public health burden of arsenic exposure in Bangladesh.

**Significance Statement**

Arsenic exposure through drinking water remains a massive public health burden in Bangladesh with an estimated 30 million people drinking water with unsafe levels of arsenic. The majority of affected residents are in rural Bangladesh, where water supply is highly decentralized. This study investigated the benefit of an inexpensive informational intervention through a randomized control trial study design. Analysis of arsenic exposure before and after the intervention indicates a clear benefit and causal relationship to the observed change. The approach is cheaper and more actionable compared to installing new deep tube wells or piped water supply systems. Consequently, well testing and informational campaigns should become part of existing and future arsenic mitigation efforts in Bangladesh.

## Introduction

Chronic exposure to arsenic can cause numerous adverse health effects (1–6). Despite more than two decades of mitigation efforts in Bangladesh, overwhelming public health and economic burdens remain (7, 8). As per the most recent multiple cluster indicator survey conducted by the United Nations Children’s Fund (UNICEF) and the Bangladesh Bureau of Statistics (BBS), more than 16% of households in Bangladesh (∼30 million people) drink water with arsenic levels above the World Health Organization (WHO) guideline of 10 µg/L, and an estimated 10.6% of households (∼17 million people) drink water with arsenic concentrations above the Bangladesh drinking water standard of 50 µg/L (9).

Interventions for environmental governance can be broadly classified into informational, incentive, and institutional approaches (10). While informational interventions are typically a less expensive approach to address existing drinking water exposure concerns, empirical support for their effectiveness in promoting behavior change is mixed (11, 12). Awareness raised by informational interventions about potential detrimental health impacts can have a strong and similar effect to wealth on the demand for improved environmental quality (13). However, due to the spatial heterogeneity of well water arsenic concentrations (14) and because arsenic contamination is unobservable without testing (i.e., arsenic has no taste, odor, or color), it is expected that the social learning processes will be slow (15). Aziz et al. report that access to arsenic awareness alone, without individual household water quality testing data, did not significantly affect households likelihood to adopt avoidance measures (16). Providing source specific water quality information, however, has been correlated with changes in drinking water source. Following several well testing efforts facilitated by the Bangladesh Arsenic Mitigation Water Supply Project (BAMWSP) between 2000 and 2004, 29% of households with arsenic levels over 50 µg/L in their drinking water well shifted their drinking water source (17). Opar et al. reported that in Araihazar, Bangladesh, when source water quality information in addition to arsenic awareness messaging were provided, 65% of 6,500 tube well users who learned their well was unsafe changed to another well within one year, while only 15% of those with safe well water at the start of the study changed sources (18). Madajewicz et al. further suggested a causal relationship between information provided about water quality and household behavior in the Araihazar study group with higher rates of well switching in households that have high levels of arsenic (12). A more recent analysis in Araihazar by Jamil et al. demonstrated the cost-effectiveness of an informational intervention in comparison to other intervention options (19). These observations suggest that the provision of awareness and well-specific water quality information can result in positive behavior change, but a causal relationship has not yet been established through a randomized control trial (RCT) design.

Accurate testing of drinking water sources is paramount. Opar et al. reported that when arsenic-safe wells were either mislabeled by BAMWSP or unmarked, nearly two-thirds of households installed new wells, abandoning safe wells for potentially unsafe wells (18). While laboratory-based measurements remain the gold standard, studies have shown that careful selection and use of inexpensive field test kits can also produce acceptable results (19–21). Fee-based testing could offer a sustainable solution to the unmet need for water testing. George et al. showed through a RCT that household education about the health effects of arsenic could increase the demand for fee-based testing (22). In their study, households were offered arsenic testing by the Econo–Quick field kit at USD 0.28 per test, accounting for roughly 20% of the total cost of administering the intervention. Household education and a local media campaign both increased the demand for fee-based arsenic testing. George et al. also showed in another RCT that testing by a community member as opposed to an external member did not lead to a significantly different outcome in terms of well switching (23). Further, in the case of arsenic-removing filters, testing must be done frequently to characterize filter performance changes over time.

Identifying an alternate safe drinking water source is the next logical step for households drinking contaminated water, but this is not as straightforward as it may seem. Variables such as water source location, water source type, and social factors can influence selection, and the questionable accuracy of prior arsenic testing compounds the complexity of making a safe selection (20). Households typically prefer tube wells as an alternate water source because of their simplicity of operation, the uninterrupted availability of water, and the almost negligible maintenance (24, 25). Consistent with these observations, Aziz et al. reported that people were willing to walk long distances to avoid exposure if the source for arsenic-safe water was a tube well, but would not if the source was surface water (16).

It is important to establish if and when information alone can induce households to seek safe water using their own resources, and to measure not only whether a change was made, but also whether this change lowered arsenic exposure, for example, by conducting a follow-up analysis of drinking water quality or by measuring urinary arsenic levels (26, 27). The current study was designed by building on prior observational studies (17, 18), with a unique intervention design consisting of providing water quality results, a personalized recommendation for alternative water sources with improved quality, reminders to the household, and assistance with further testing at no cost if needed. We hypothesized these aspects of the study improved the likelihood that households would change to a water source of better quality, and the quantum of this change was measured in our follow-up visit post intervention. We used a RCT design to quantify and establish the benefit of the informational intervention. The approach can be applied in other arsenic-affected areas of Bangladesh.

## Results

### Baseline results and study design

Our study was carried out in Phulsara Union, Chowgacha Upazilla, Jessore District, Bangladesh. Due to the hydrogeology of this area, deep tube wells (>150 m) are not typically a feasible safe water alternative. Therefore, arsenic mitigation strategies in the region rely on arsenic removal treatment technologies and surface water treatment technologies. Specifically, only 15% of 94 safe water devices (SWDs) installed in Phulsara Union consisted of deep tube wells (28). This observation stands in sharp contrast with national statistics, which indicate that 84% of SWDs installed in arsenic-affected regions are deep tube wells (29).

At the baseline survey, water from 127 households tested below 10 µg/L, while the remaining 354 households formed our study group. The mean arsenic level across 481 household water sources tested at the baseline was 107 µg/L while 50% of the samples tested below 50 µg/L. According to the BAMWSP data for Phulsara Union from 2005 [compiled data accessed from the Supplementary Information of Jamil et al. (19)], the mean arsenic level across 2,213 wells tested then was 69 µg/L and 46% of the wells had arsenic levels below 50 µg/L.

**Fig. 1.**
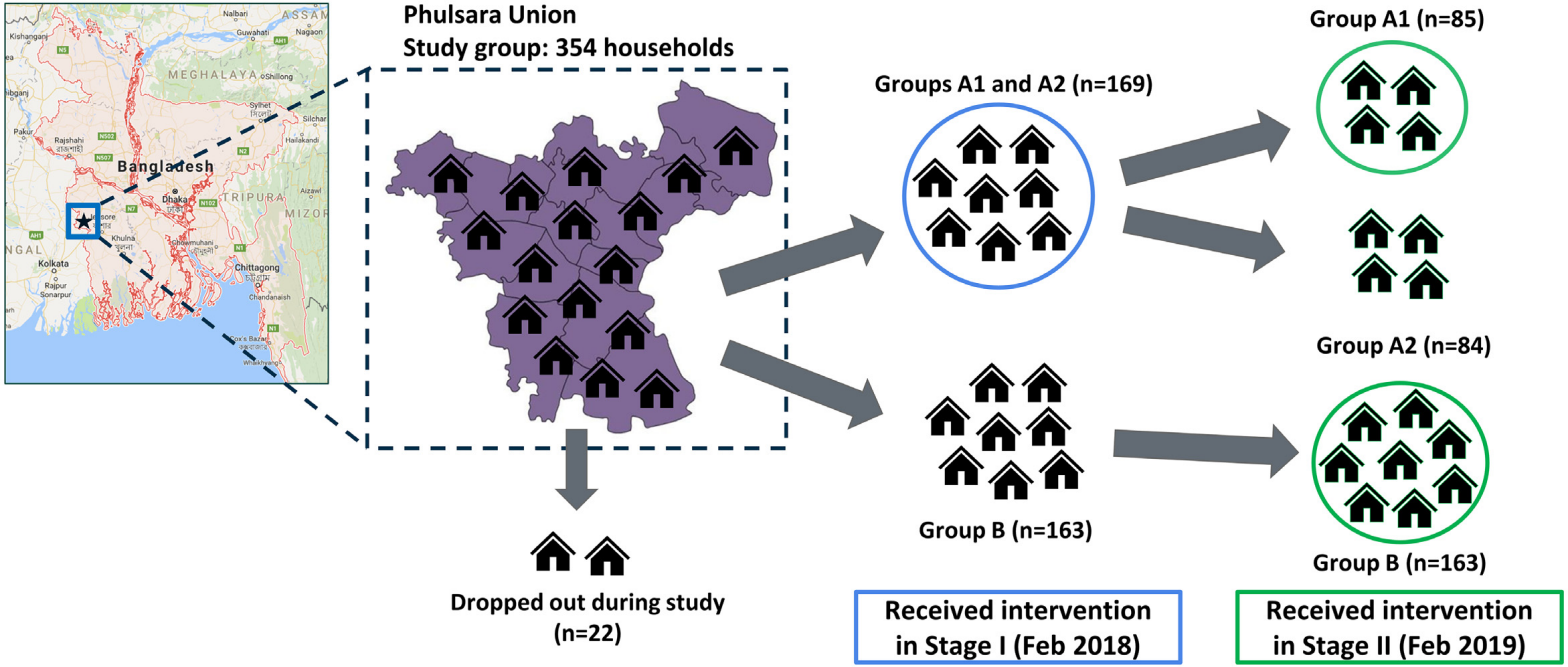
Study design and study groups: The study was conducted in Phulsara Union (∼6,500 households total) in southwestern Bangladesh. Of the 354 households whose water tested above the WHO guideline for arsenic (10 µg/L), 22 dropped out over the study duration. Intervention Stage I was applied in February 2018 to Group A, but not to Group B. Midline measurements were made in March 2018 for both Group A and Group B. Intervention Stage II was applied in February 2019 to Group A1 and Group B, but not to Group A2. Endline measurements were made in March 2019 for all households remaining in the study.

After identifying households whose water tested above the 10 µg/L WHO guideline for arsenic in the baseline survey, we implemented a staggered intervention design (Fig. 1) that ensured all selected households received an informational intervention over the course of the study. The informational intervention provided three elements: (i) educational materials on arsenic as a drinking water contaminant, its long-term adverse health effects, and the Bangladesh standard and WHO guideline for arsenic in drinking water (Supplementary Figs. S1 and S2); (ii) the arsenic test results of the household drinking water source sample collected during a household survey and summarized for the user in the context of the Bangladesh standard and WHO guideline (Supplementary Figs. S3 and S4); (iii) recommendations for alternate safer drinking water sources (both private and public) for the household based on our database of sources in the vicinity of that household (Supplementary Fig. S5).

### Response to informational intervention

**Fig. 2.**
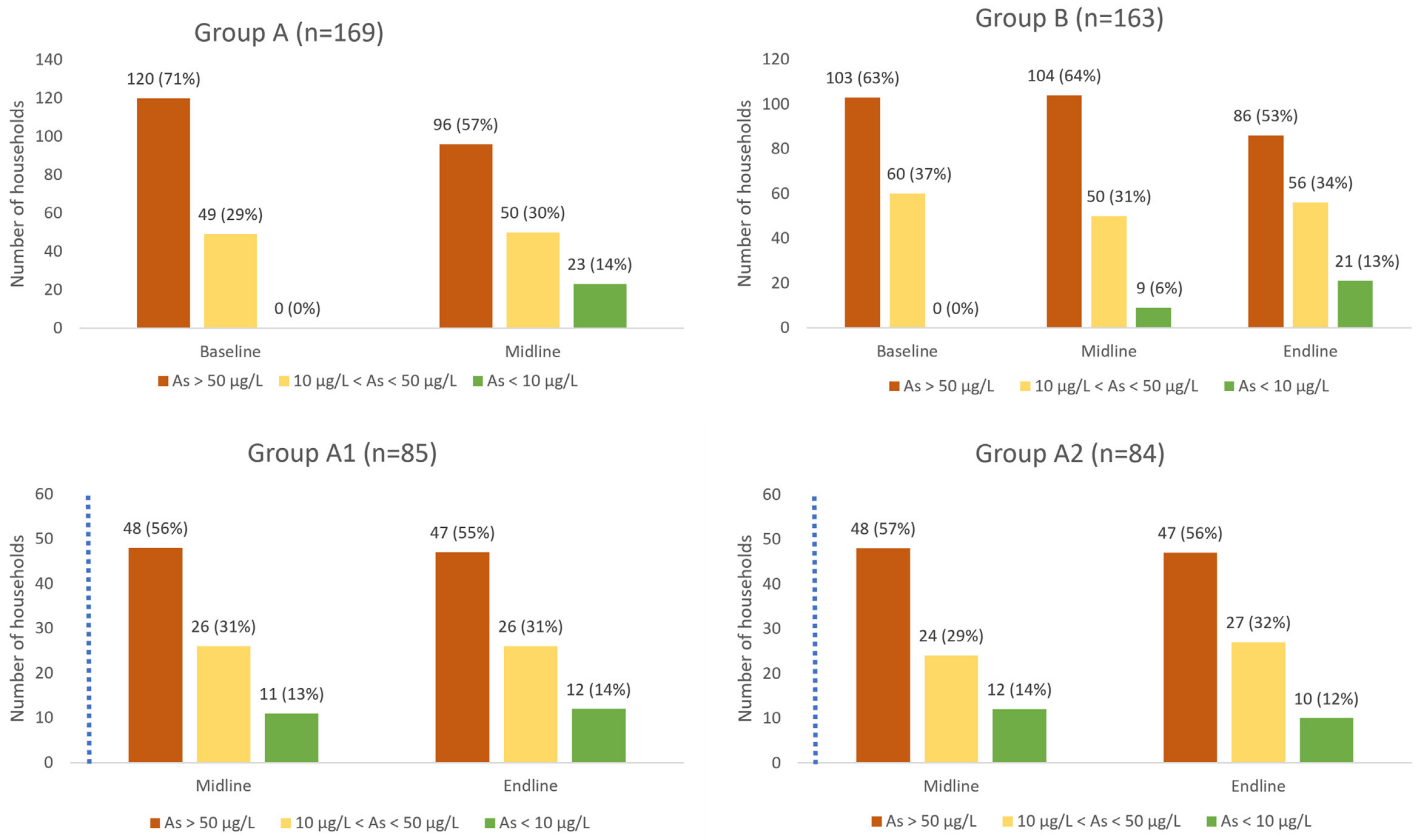
Households with drinking water arsenic levels below the WHO guideline (<10 µg/L), above the WHO guideline but below the Bangladesh standard (10 to 50 µg/L), and above the Bangladesh standard (>50 µg/L) across the intervention. A single dotted blue line represents an intervention at Stage I; two dotted blue lines represent an intervention at Stage II. The bars to the left of the blue dotted line in the panels represent numbers before the intervention, while the bars on the right represent numbers after the intervention (a) Group A household arsenic concentrations before (left) and after (right) Stage I intervention. (b) Group B household arsenic concentrations at all three measurement timepoints. Group B received Stage II intervention between the midline and endline. (c) Group A1 household arsenic concentrations before (left) and after (right) receiving the second intervention at Stage II. (d) Group A2 household arsenic concentrations at midline and endline measurements, which were both after the Stage I intervention. Group A2 did not receive the second intervention at Stage II. However, arsenic concentrations at equivalent timepoints provide a reference to assess the effect of the second intervention on Group A2.

In Stage I of the study, 37% of households who received the intervention (Groups A1 and A2) reported changing their water source or treatment, while only 10% in Group B reported making this change. 32% of households that received the intervention accepted the offer of testing an additional water source at no cost to them and, among this subset of households, 53% had changed their water source. Pairwise *t*-test comparisons showed that arsenic concentrations declined significantly in the drinking water sources of the households that received the intervention (Groups A1 and A2, *P* = 0.0002), but remained unchanged in households that did not receive the intervention (Group B, *P* = 0.93). Specifically, the number of households with drinking water arsenic concentrations above 50 µg/L in Groups A1 and A2 declined from 120 (71%) at baseline to 96 (57%) at midline and remained nearly identical in Group B with 103 (63%) at baseline and 104 (64%) at midline (Fig. 2).

In Stage II of the study, 33% of households in Group B reported changing their water source or treatment after receiving the intervention. 28% of households in Group B accepted the offer of testing an additional water source at no cost to them and, among this subset of households, 55% had changed their water source. We observed a decrease in the number of households whose drinking water tested above 50 µg/L from 104 (64%) at midline to 86 (53%) at endline (Fig. 2). However, the decrease was not statistically significant (Group B, *P* = 0.17) in a pairwise comparison of arsenic levels. In Group A1, 47% of households reported changing their water source or treatment after receiving the intervention. A total of 27% of households in Group A1 accepted the offer of testing an additional water source at no cost to them and, among this subset of households, 74% had changed their water source. We did not observe a further decrease in drinking water arsenic levels for households that had received the intervention a second time (Group A1) (*P* = 0.39). Neither was a decrease observed for households that had not received the intervention a second time (Group A2) (*P* = 0.77) (Fig. 2).

The distribution of arsenic concentrations in household drinking water sources across study groups at the three measurement timepoints (baseline, midline, and endline) is summarized in Table 1. The mean arsenic concentrations at baseline in Group A and Group B were similar (149 ± 115 µg/L and 138 ± 122 µg/L, respectively). After Group A received the intervention, the mean arsenic concentration decreased to 118 ± 138 µg/L, while it remained similar for Group B at 139±147 µg/L, indicating the benefit of receiving the intervention.

**Table 1.** Distribution of arsenic concentrations in household drinking water sources across households in each study group.

		% of households within group with arsenic concentration				Arsenic concentration (µg/L)		
		<10 µg/L	10–50 µg/L	>50 µg/L	>150 µg/L	Mean	Stdev	Median
Group A (*n* = 169)	Baseline	0	29	71	49	149	115	143
	Midline	14	30	57	31	118	138	60
	Endline	13	31	56	36	125	128	62
Group B (*n* = 163)	Baseline	0	37	63	42	138	122	106
	Midline	6	31	64	36	139	147	90
	Endline	13	34	53	34	123	136	53
Group A1 (*n* = 85)	Baseline	0	27	73	47	143	113	138
	Midline	13	31	57	25	104	126	60
	Endline	14	31	55	32	114	125	60
Group A2 (*n* = 84)	Baseline	0	31	69	50	155	117	148
	Midline	14	29	57	37	133	149	61
	Endline	12	32	56	39	136	131	75

Given the high variance in the concentrations, which ranged from below the detection limit (0.7 µg/L) to 650 µg/L, the median values (Table 1 and Supplementary Table S3) offer additional insight into the effect of the intervention. The median arsenic concentration decreased substantially from 143 to 60 µg/L in Group A following the intervention, while the median arsenic concentration in Group B had only decreased slightly from 106 to 90 µg/L. Following the Stage II intervention, the median arsenic concentration in Group B decreased from 90 to 53 µg/L. At endline, the median concentration in Group A2, which had not received the Stage II intervention, had increased from 61 to 75 µg/L, whereas the median concentration in Group A1, which received the Stage II intervention, remained constant at 60 µg/L.

Regression analyses, while controlling for water source type and a proxy for household income (electricity), two possible confounding variables, showed that households in Group A that had received the intervention at Stage I had arsenic levels in their drinking water that were, on average, 64% lower than households in Group B that had not received the intervention (*P* < 0.001). A similar analysis after Stage II, indicated that households in respective Groups A1, A2, and B had, on average, 63, 61, and 59% lower arsenic levels in their drinking water than at the beginning of the study (*P* < 0.001). At the end of the study, there was no significant difference between the responses in Groups A1 and A2 (*P* = 0.83), Groups A1 and B (*P* = 0.71), and Groups A2 and B (*P* = 0.90), indicating that all study groups responded similarly to the intervention.

The primary mechanism for reduction in household drinking water arsenic levels involved households making an informed change in their drinking water source. Households that received the intervention were more likely to change their water source, and this change was more likely to result in lower arsenic exposure. Across Stage I of the study, households that received the intervention (Group A) were 3.7 times more likely to change their drinking water source or implement water treatment when compared to households that did not receive the intervention. Of the 63 households in Group A that changed their drinking water source after receiving the intervention, water quality improvements were significant (*P* < 0.0001). Specifically, their mean arsenic concentrations decreased from 170 to 76 µg/L and their median arsenic concentrations decreased from 167 to 41 µg/L. In contrast, for the 17 households in Group B that changed their drinking water source without receiving the intervention during Stage I, the improvements in water quality were not statistically significant (*P* = 0.11), although their mean arsenic concentrations decreased from 144 to 98 µg/L and their median arsenic concentrations decreased from 162 to 90 µg/L. For the intervention households that did not report changing their drinking water source (*n* = 107), the change in arsenic levels was not significant (*P* = 0.50). These observations suggest that households that received the intervention and responded by changing their water source were able to make informed drinking water choices (i.e., change to a safer drinking water source) and reduce arsenic exposure.

When considering only the subset of Group A households that were below 150 µg/L [a threshold for high arsenic based on findings of prior studies (12, 27)] at baseline, no significant difference in arsenic levels was observed after the intervention (*P* = 0.39).

Shallow tube wells are associated with higher drinking water arsenic levels across the study population. In comparison to households drinking water from a shallow tube well and controlling for the intervention, households drinking water collected from an arsenic iron removal plant, deep tube well, ring well, and dug well with a sand filter had, on average, 52% lower (*P* < 0.001), 60% lower (*P* < 0.001), 77% lower (*P* < 0.001), and 86% lower arsenic levels (*P* < 0.002), respectively. Also of note, having electricity (a proxy for household income level) was not a significant predictor of household drinking water arsenic level (*P* = 0.94).

To understand whether household treatment or storage methods post collection affected arsenic concentrations between collection and consumption, we collected a sample of drinking water at the source and an additional sample of drinking water at the household for all households at midline. No significant difference between arsenic levels in the two sets of samples was found (*P* = 0.34). Supplementary Fig. S6 shows the arsenic levels in these household and source water samples taken at the midline stage.

## Discussion

This study demonstrated that a low-cost informational intervention led to a substantial number of households reducing their arsenic exposure by changing their drinking water source. Further, we believe that the efficacy of this approach will improve if a higher fraction of water sources are tested (in our study, about 10% of water sources were tested), as this will identify all safe water sources and enable users to make the most informed choice. Our findings build on past observational studies suggesting the benefit of information and well testing schemes (12, 17–19, 27). Further, we found that while receiving the intervention a second time led to more households changing their water source, it did not result in any additional lowering of arsenic levels. This could improve with more testing but also indicates that information alone does not address all the barriers to ensuring arsenic-safe drinking water. Bolderdijk et al. argue that informational interventions are effective when the behavior change is easy, convenient, or not costly (11). Opar et al. suggest that distance to an alternate water source impacts the likelihood of changing and that additional barriers could include the social costs of sharing a private or communal water source (18). Madajewicz et al. report well sharing is much more common among relatives and suggest that people are reluctant to use the well of a nonrelative (12). Future work should identify these barriers and how they can be addressed.

We measured changes in drinking water source arsenic concentrations as a proxy for household arsenic exposure, which studies measuring urinary arsenic have shown is a good indicator of arsenic exposure in the rural Bangladeshi context (26, 30). Some households reported treating water post collection, such as allowing water to settle in a bucket and discarding the precipitate. Since arsenic levels in water samples collected at the drinking water source and at the household did not differ significantly (*P* = 0.34), we conclude such practices, on average, did not reduce arsenic levels. We occasionally experienced some difficulty in identifying a household’s primary drinking water source, with over 55% of households at the endline, particularly those using tube wells, using more than one drinking water source during part of the year. We relied on the key informant to define the primary drinking water source for the sample collection, but note that alternate sources of water could be a significant part of an individual’s water consumption. Huhmann et al. (31) estimate that in the Health Effects of Arsenic Longitudinal Study (HEALS) study population in Araihazar, individuals consume 25 to 40% of their water from water sources other than their primary source, with self-reported survey data indicating that on average women consume 91% of their water from the primary source, while for men it is lower at 70%. Further, although not considered in the present study, food may also be a significant source of arsenic exposure for households (32), particularly rice and seafood, and thus remain an important effect to evaluate. We also note that studies that have reported measuring urinary arsenic levels have indicated that arsenic concentrations in primary household water alone might underestimate arsenic exposure at the low end (in households with As < 10 µg/L in the primary water source) and overestimate arsenic exposure at the high end (>150 µg/L) due to the relative contributions of other water sources in each case.

While arsenic is the primary contaminant of concern in groundwater-based water sources in Bangladesh, it is not the only one. Microbial contaminants have been found seasonally in groundwater (9, 33). Further, high levels of salinity (common in the coastal belt), iron, and manganese pose aesthetic issues and impact water acceptability (25, 34). While this study did not investigate water quality contaminants besides arsenic, future work should do so to determine the suitability of alternative water sources for drinking and cooking.

Across study groups, about a third of the households receiving the intervention accepted our offer of testing an additional water source for free, and among these households we saw a higher fraction changing their water sources (53, 55, and 74, respectively). This suggests the benefit of providing easy and accessible testing. Furthermore, our observation that the subset of households with arsenic levels <150 µg/L did not respond significantly to the intervention suggests that an arsenic threshold exists above which households are more likely to address water quality concerns. This finding is consistent with results reported by Madajewicz et al. (12) and Huhmann et al. (27) Additionally, our study has shown that an informed choice improves the likelihood of changing drinking water sources to a source with better water quality when households are provided with information about nearby alternate safe water sources. Based on these observations, we suggest creating water supply maps (Supplementary Fig. S5) for informational campaigns in concert with well testing campaigns. This creates an effective integration of past and new intervention approaches and provides compounding benefits for improved water quality for arsenic-affected communities.

A 2012 report based on two independent studies estimated that arsenic exposure at 2009 levels was resulting in 43,000 to 56,000 additional deaths per year and would result in USD 13 billion in lost productivity across a 20-year time period (8). The average cost of the intervention evaluated in our study (including materials, arsenic analyses, field worker wages, and transportation) was less than USD 10 per household (Supplementary Table S1). Laboratory analyses accounted for approximately half the intervention cost, and this could be lowered by 30 to 40% if certain field test kits were used for arsenic measurements instead, without significantly compromising accuracy (20). A recent study by Jameel et al. indicated that, in the context of Bangladesh, the benefits of using accurate laboratory data, as opposed to less accurate data collected with field kits, are too modest to justify the additional expenses (19). While 79% of households in our study indicated that they would be willing to pay BDT 150 (about USD 1.6) for an arsenic test, a fee which would cover field kit supplies plus additional costs to administer it at a household level, prior studies in Sonargaon, Bangladesh (35) and Bihar, India (36) reported that only 25 to 30% of households opted for a fee-based test priced around USD 0.6 pointing to the likely gap between stated and actual willingness to pay for a test. Assuming a conservative estimate of USD 6 per household for the intervention by using field test kits (Supplementary Table S1), such an effort would cost between USD 40 and 230 million to administer across the estimated 7 million Bangladeshi households drinking water with arsenic levels >10 µg/L out of ∼38 million households overall (9, 37).

Due to the highly decentralized nature of water supply in Bangladesh—with <12% of the population using piped water (9)—setting up water treatment and distribution infrastructure would be a massive economic and political undertaking. While it could be argued that such informational interventions are a stopgap measure (38) and much work remains to transition to a long-term solution that ensures sustainable, safe drinking water for all Bangladeshis, in the interim such efforts can provide enormous public health benefits. Jamil et al. demonstrated that well testing and informational campaigns can provide immediate benefits at a fraction of the cost of installing new intermediate wells, new deep tube wells, or a piped water system (19). The low cost of such an informational intervention and its clear effect in reducing arsenic exposure through drinking water make a compelling case for an expansion of such interventions in Bangladesh. This work suggests a set of integrated and actionable intervention approaches to arsenic exposure mitigation that donors and stakeholders should consider in efforts to combat the crippling public health burden created by drinking water contamination.

## Methods

### Study site, outcome measures, and implementation

Phulsara Union consists of roughly 6,500 households across 16 villages. We selected this union due to the high incidence of arsenic contamination in shallow tube wells and the community presence that our NGO partner Asia Arsenic Network has there. Community water sources used in the area include deep tube wells, arsenic and iron removal plants, ring wells, and dug wells with sand filters. The concentration of arsenic measured in household drinking water samples was the key outcome measure of interest. Secondary outcome measures included: choice of drinking water source and awareness levels about arsenic as a drinking water contaminant.

Measurements were made at baseline (August 2017), at midline (March 2018) following Stage I intervention, and endline (March 2019) following Stage II intervention. At each measurement stage, members of the study team visited all 94 designated community SWDs in Phulsara Union to assess existing functional status and collect a water quality sample. The GPS locations of all households, water sources, and community SWDs were recorded using handheld Garmin eTrex 10 units (Garmin, USA). We collected survey data on electronic tablets using the Qualtrics survey platform. The survey can be viewed at https://doi.org/10.7302/955e-0877. Survey administrators were university students local to Phulsara Union who had prior experience with survey data collection and spoke English and Bangla fluently.

The intervention was administered by community workers who were recruited from within Phulsara Union and trained by the University of Michigan and Asia Arsenic Network study teams. The study team made efforts to ensure that the community worker who visited a household was from the same ward.

### Informational intervention design and materials

Every 12th household was visited in each of the 16 villages in Phulsara Union for the baseline survey, and this resulted in a baseline sample size of 481 households yielding a sample size of about 10% of all households in the Union. If a particular household declined to participate or was not available, the survey team moved to the adjacent household. In this way, we were able to randomly sample households across the entire union. Drawing from the baseline survey results of these initial 481 households, we provided the 127 households that tested below 10 µg/L for arsenic with educational materials and their household water quality test results; however, these households were excluded from the study thereafter. The remaining households that had tested above 10 µg/L (*n* = 322 after 22 households dropped out or were removed due to incomplete data collection during the study) were assigned randomly to one of two groups (Fig. 1): Group A (*n* = 169) and Group B (*n* = 163). Group A was split randomly into Group A1 (*n* = 85) and Group A2 (*n* = 84). Similar demographic characteristics across the three study groups (Supplementary Table S2) indicated satisfactory randomization while creating the groups. The entirety of Group A (both A1 and A2) received the intervention at Stage I, while only Group A1 received the intervention at Stage II. Group B received the intervention at Stage II only. In this way, all households received the intervention over the course of the study, with Group A1 households receiving it twice. We collected measurements across all groups approximately four weeks after each intervention began. Randomization was done using the list of household IDs (HHIDs) and a randomizing function on Microsoft Excel.

Following the baseline, midline, and endline measurements, drinking water source quality was classified into three categories—As < 10 µg/L, 10 < As<50 µg/L, and As>50 µg/L—based on the current WHO guideline (10 µg/L) and the Bangladesh standard (50 µg/L) for arsenic concentrations in drinking water. We prepared test result certificates for each household receiving the intervention with their most recent water quality result (reported to the nearest μg/L) printed on a certificate. The certificate was coded in green, yellow, or red based on the three respective categories above. In addition, certificates included corresponding messaging that aided in the interpretation of the arsenic test result. Example certificates in Bangla and their English translations can be seen in Supplementary Figs. S1 to S4. We made efforts not to overemphasize the thresholds for these classifications and to appropriately contextualize the results. Thus, while a test result of 52 and 48 µg/L would be classified in different brackets because of respectively being slightly above and below the 50 µg/L threshold, the messaging for each of these households would have been similar, and both would have been encouraged to switch to a water source with lower arsenic levels if possible. Along similar lines, while test results of 52 and 252 µg/L would both be given a red certificate for being >50 µg/L, field staff would have emphasized the need to switch to a water source with lower arsenic levels more for the latter.

We created a set of maps for each village using the Q-GIS software (https://www.qgis.org), with marked locations of each household and community water point surveyed, together with information about the level of arsenic contamination at each site (see Supplementary Fig. S5 for an example). We also prepared datasheets with additional information (e.g., GPS coordinates, water source type, water quality data, landowner information) that would help physically locate each of the points on the map. Field staff relied upon these maps and data sheets while administering the intervention.

The three components of the intervention (educational material, an arsenic test result, and a recommendation for alternate sources of water) were provided to all household members present during a household visit. A test result certificate was delivered directly to the head of the household only. A follow-up visit was made if the head of the household was not present. Approximately one week after the household visit, the study team made a follow-up phone call to the head of household to remind them of the three components of the intervention and to answer any questions. Households were offered water quality testing free of charge if they had switched to a water source that had not already been tested by the study team or were considering switching pending water quality testing. Approximately one week after this phone call, the study team sent a follow-up text message (to the head of household and any other phone numbers associated with the household that were provided by the key informant) with an additional reminder of the three components of the intervention. Interventions at Stage I and Stage II were identical in their delivery.

### Arsenic measurement

We collected water samples directly from the primary drinking water source identified by the household member surveyed (the key informant). For shallow and deep tube wells, a minimum of 50 and 100 presses of the hand pump, respectively, were made before sample collection to ensure collection of water from the aquifer rather than from water standing in the tube well (21, 39). For water treatment systems, the treated water was sampled directly. The water samples were collected in 125 ml plastic containers that were acid-washed in the laboratory before field collection. The containers were rinsed three times with the sample water before collecting ∼100 ml and capping the container. Once returned to the laboratory, samples were acidified with 6 N hydrochloric acid to a final concentration of 2% (v: v) and analyzed with Hydride Generation Atomic Absorption Spectroscopy (HG-AAS; Shimadzu, Japan) using standard protocols (40). The method detection limit was determined to be 0.7 µg/L. The instrument was set to allow a maximum relative standard deviation (RSD) of 5% between triplicate absorption reads. Daily calibrations were performed, as well as a standard check once every ten samples, allowing us to reject and repeat any analyses where the RSD on the standard check varied by more than 5%. A calibration curve was generated daily, and if the internal standard (run every ten samples to check for instrumental drift) varied by more than 10% the system was recalibrated, and samples were re-run. Recoveries of standard additions to distilled water and groundwater sample matrices were between 80 and 120%. A random selection of samples was sent to a commercial laboratory (Bangladesh Council of Scientific and Industrial Research-BCSIR, Dhaka, Bangladesh) for cross-laboratory verification and showed consistent results with RSD between the sets of <9%.

### Statistical analyses

A longitudinal analysis of outcome measures before and after intervention stages was conducted. The significance levels reported are based on a pairwise t-test of arsenic levels at the two corresponding time points and are two-sided.

A linear mixed effects regression model using the “lmer” function in the “Linear Mixed-Effects Models (lme4)” software package in R (https://www.rstudio.com/) was also used on the same dataset to model the household arsenic levels after the intervention as a function of the arsenic level prior to the intervention. This was done twice, corresponding to each intervention stage. The independent variables considered in the regression model included: whether the household received the intervention, the drinking water source type, and whether the household had electricity (which served as a proxy for household income). Random effects considered in the model were HHID and village name, which reduced redundancy. We reported two-sided P value estimates based on the model.

## Data Availability

The data associated with this study are available at https://doi.org/10.7302/bqwn-m402.

## Code Availability

The code used in the R software product for the regression model that supports the findings of this study is available from the authors on request.

## Author Declaration

M.J.A and M.R.K are employees of Asia Arsenic Network, a non-profit organisation involved in arsenic mitigation efforts in Bangladesh. AAN has received funding from and worked on several projects with international and local agencies including the Japan International Cooperation Agency (JICA), United Nations Children's Fund (UNICEF) and the Bangladesh Department of Public Health Engineering (DPHE).
